# The impact of two multiple-choice question formats on the problem-solving strategies used by novices and experts

**DOI:** 10.1186/1472-6920-4-23

**Published:** 2004-11-05

**Authors:** Sylvain P Coderre, Peter Harasym, Henry Mandin, Gordon Fick

**Affiliations:** 1 Department of Medicine, University of Calgary, Health Sciences Centre, 3330 Hospital Drive NW, T2N 4N1. Calgary, Alberta, Canada; 2 Department of Community Health Sciences, University of Calgary, 2500 University Drive NW, T2N 1N4. Calgary, Alberta, Canada; 3 Division of Nephrology, Foothills Hospital. 1403 29^th ^St. NW, T2N 2T9. Calgary, Alberta, Canada

## Abstract

**Background:**

Pencil-and-paper examination formats, and specifically the standard, five-option multiple-choice question, have often been questioned as a means for assessing higher-order clinical reasoning or problem solving. This study firstly investigated whether two paper formats with differing number of alternatives (standard five-option and extended-matching questions) can test problem-solving abilities. Secondly, the impact of the alternatives number on psychometrics and problem-solving strategies was examined.

**Methods:**

Think-aloud protocols were collected to determine the problem-solving strategy used by experts and non-experts in answering Gastroenterology questions, across the two pencil-and-paper formats.

**Results:**

The two formats demonstrated equal ability in testing problem-solving abilities, while the number of alternatives did not significantly impact psychometrics or problem-solving strategies utilized.

**Conclusions:**

These results support the notion that well-constructed multiple-choice questions can in fact test higher order clinical reasoning. Furthermore, it can be concluded that in testing clinical reasoning, the question stem, or content, remains more important than the number of alternatives.

## Background

The assessment of problem-solving skills, and specifically diagnostic skills, was once reserved for examination formats such as free-response questions, patient management problems (PMPs) or oral examinations. These evaluation methods, however, are all resource-intensive, thus making it difficult to provide the representative sampling of problems necessary to circumvent the problem of case specificity, which predicts that success in solving one clinical presentation does not predict success in another [1]. As a consequence of case specificity, reliability and content validity of an examination are dependent on a broad sampling of problems. Such extensive sampling is more easily done with pencil-and-paper type of tests. This study will examine two pencil-and-paper formats specifically in regards to their relative problem-solving testing abilities.

Previous literature has demonstrated that altering item stems tends to determine clinical challenge, while psychometric properties such as discrimination and difficulty tend to be affected by the number of answer options [2], hereby referred to as 'number of alternatives'. The central focus of this paper surrounds whether altering the number of alternatives within a pencil-and-paper format alters diagnostic higher order thinking and/or format psychometric properties. Two formats were studied, both with a stem consisting of a long vignette with distracters, but with different number of alternatives. The format presenting five options to the examinee will henceforth be referred to as the "multiple-choice question" or MCQ format, while the second format, presenting greater than ten options to the examinee, will be referred to as "extended-matching" or EMQ format.

The first examination format studied is the five-option MCQ (see Appendix A for example). Although MCQs have always been considered an efficient and reliable testing tool, they have not always been perceived as ideal for the evaluation of higher-order thinking skills such as problem solving. Prevailing perceptions that MCQs assess lower levels of knowledge such as recall of isolated facts, and/or encourage trivialization, do exist in the medical education community [3]. To the extent that some clinicians question whether MCQs can test problem-solving skills, suggests that this format may have low validity [4]. However, as discussed by Case and Swanson [5] well constructed MCQs could challenge students to problem solve. Maguire et al also recognized that MCQs could yield valid information of clinical reasoning skills, providing that stems and alternatives are well constructed [6]. Evidence does exist that MCQs have predictive value for more recognized problem-solving tasks [7] and can elicit higher order problem solving such as forward reasoning [8].

The second examination format the EMQ format, initially designed in response to some of the criticisms of the MCQs. EMQs (see Appendix A for example) were introduced in the 1990s in both the NBME and USMLE, amongst others. Case and Swanson [5] have been instrumental in the development of these questions, which are defined as any matching format with more than the five alternatives traditionally used by MCQs. From its conception, the question preparation of the EMQs has been very careful in designing stems that test higher cognitive levels such as problem solving. The first study that examined the psychometric features of Extended-matching [5] questions showed that Extended-matching items were more difficult, more discriminating, had higher reliability and needed significantly less testing time to achieve reproducible scores than traditional MCQs. Other studies have shown that EMQs, by increasing the number of alternatives used, increased mean item difficulty as well as, perhaps by reducing guessing, provided improvement in item discrimination over the five-option MCQ [9]. By increasing item discrimination, EMQs offer comparable levels of reproducibility with 30% fewer items than the MCQ with five options [9]. Reliability coefficients were also markedly higher with Extended-matching [5]. Positive psychometric outcomes have been found in other studies using the format [10-14].

These studies have focused on psychometrics, whereas potential benefits, and possible reasons for such benefits, of the EMQ format over standard MCQs in eliciting higher order problem solving remain unclear. No study has formally used think-aloud protocols to assess whether a well-written MCQ differs from EMQs in challenging examinees to problem solve. There is little doubt that poorly written MCQs can encourage students to learn isolated facts by rote. In fact, all available evaluation methods potentially yield information on clinical reasoning if the content is appropriate, suggesting that content is more important than question type [15].

The two examination formats will be tested for their ability to elicit the three different diagnostic reasoning strategies generally available to learners: hypothetico-deductive reasoning, pattern recognition, and scheme-inductive reasoning. Deductive reasoning (hypothetico-deductive) [16] is a "to-and-fro" strategy of problem solving, also termed "backward reasoning". The method is generally used by novices or experienced diagnosticians to include or exclude a single diagnosis, when faced with a particularly complex problem, or as a fallback strategy when faced with clinical problems that are outside their domains of expertise.

Pattern recognition has been identified by other research as a very successful approach used by experts to solve clinical problems [17-19]. Before becoming more expert in problem solving, learners progress through several transitional stages characterized by different knowledge structures: elaborated causal networks, abridged networks, illness scripts, and instance scripts [18]. Extensive experience eventually leads to acquisition of a repertoire of problems common to the domain of expertise termed "illness scripts". This repertoire permits problem resolution by recognition of new problems as ones that are similar or identical to old ones already solved, and the solutions are recalled.

**Figure 1:**
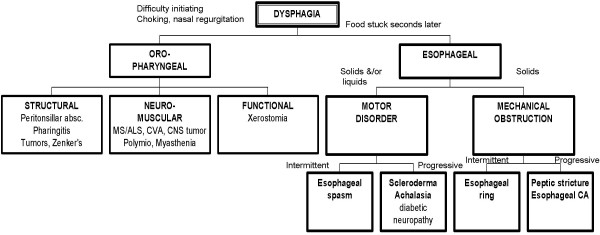
Example of the scheme for "dysphagia".

The third strategy is scheme-inductive reasoning. "Schemes" are defined as a mental categorization of knowledge that includes a particular organized way of understanding and responding to a complex situation. They are drawn on paper like "inductive trees" or "road maps" to recreate the major divisions (or chunks) used by expert clinicians for both storage of knowledge in memory and its retrieval for solving problems [19,20] (see Figure 1 for an example of the scheme for "dysphagia"). Decisions are explicitly at the forks in the road or branching of the tree. The organizational structure, or "scheme", proceeds from alternative causes in a forward direction, through crucial "tests", to exclusion of some alternative causes and adoption of what is left. These tests may be based on an evaluation of symptoms, signs, or results of investigations, singly or in any combination. Scheme-inductive reasoning is a strategy used by experts when pattern recognition is not possible [21]. This type of problem solving represents the "climbing of a conditional inductive tree" [22].

By directly comparing the problem-solving strategies elicited by the two pencil-and-paper formats, using the think-aloud method previously described, two major questions will be addressed. The first question is whether pencil-and-paper formats such as EMQ and MCQ can in fact assess problem-solving skills. The examination formats' capacity to evoke more 'expert' methods of problem solving, such as scheme-inductive reasoning or pattern recognition, will be taken as evidence of their ability to assess problem-solving skills. The second question relates to the impact of the alternatives number on psychometric properties and diagnostic higher order thinking, considering that a shift to hypothetico-deductive reasoning could conceivably occur with the shorter alternatives lists of the MCQ format. A corollary to these questions is whether in testing problem solving, it is the construction of question stems that is important, as opposed to the number of alternatives or examination format.

## Methods

### Examination construction

An examination for four clinical presentations, each representing a different domain in gastrointestinal medicine, was constructed: dysphagia, chronic diarrhea, nausea and vomiting, and elevated liver enzymes. The examination consisted of eight pencil-and-paper questions, with two questions, one of the MCQ type and another of the EMQ type, created for each of the four clinical presentations. While completing the questions, the examinees were permitted to write notes.

The two question stems written for each of the clinical presentations were long vignettes created with a problem-solving task in mind. Furthermore, the stems within each clinical presentation (see Appendix A for the two stems for clinical presentation 'diarrhea') were designed to be as similar as possible in length, difficulty, and the presence of distracters. The stems differed only in the presence of a few key different pieces of information that led to a different diagnosis. The stems were then randomly assigned to one of the examination formats described above, MCQ or EMQ. The alternatives list included the correct diagnosis, and two plausible 'competing alternatives' to the correct answer.

### Subjects

The examination was administered to twenty experts in Gastroenterology in two centers, Calgary (15) and Ottawa (5), as well as twenty non-experts, final-year medical students at the University of Calgary. Candidates were considered experts if they were specialists who spent more than 80% of their clinical time in the practice of Gastroenterology.

### Data collection

The subjects were first asked to answer the eight questions. The examinees were not given a time constraint to complete the examination, though most completed it in 45 minutes. After the completion of the eight questions, the subjects, with the examination paper in hand and any written notes made during the examination, were asked to explain how they arrived at each diagnosis. A panel of two judges (experts in the Gastroenterology presentations being tested and in the recognition of the diagnostic reasoning process) interviewed the examinees. With as little prompting as possible, the examinees were asked to think-aloud [23] and describe how each diagnosis was derived. Based on the examinees' verbal discourse for that question, the two judges assigned a discrete 'Process Score' of 1, 2, or 3, depending on the predominant diagnostic process used. Once the score was assigned, the examinee was encouraged to proceed to the next question, until a diagnostic process score had been assigned for all eight questions.

A 'Process Score' of 3 was assigned if pattern recognition was used. Determination that "pattern recognition" was used occurred when the subject directly reached a single diagnosis with only perfunctory attention to the alternatives. A 'Process Score' of 2 was assigned if a well-structured and accurate scheme was predominantly used to guide the inductive inquiry. Determination that a scheme-directed diagnostic reasoning strategy was used occurred by analysis of the verbal discourse using modified propositional analysis [24]. A proposition is defined as "the smallest unit of meaning that underlies the surface structure of a text" [25]. This analysis consisted of searching the examinees' discourse for key predetermined propositions that linked categories and thus provided evidence for chunking (i.e. scheme use). These key chunking propositions were determined by the authors based on information from texts, databases, consultation with experts not participating in the study, and personal experience. A recall method was utilized and felt appropriate given that of major interest to the present study was global description of representations in memory, as opposed to exact numbers of recall or specific inferences made from recalled texts [26]. The key propositions are shown in Table 1.

**Table 1:** Propositions demonstrating evidence of chunking.

Clinical presentation	Key chunking propositions
Dysphagia	- Oropharyngeal vs. esophageal - Mechanical vs. motility
Elevated liver enzymes	- Hepatocellular vs. cholestatic - Intra vs. extrahepatic cholestasis
Nausea and vomiting	- GI vs. non-GI causes - GI vs. metabolic vs. CNS vs. drugs
Diarrhea	- Small bowel vs. large bowel - Steatorrhea (malabsorption) vs. non-steatorrhea - Osmotic vs. secretory vs. inflammatory vs. motility

A 'Process Score' of 1 was assigned if the examinee relied on hypothetico-deductive reasoning exclusively or predominantly. It was determined that hypothetico-deductive reasoning was the diagnostic strategy utilized when the subjects analyzed one by one each alternative diagnosis presented with the clinical vignettes prior to selecting the most likely diagnosis.

The interviews were audio taped or videotaped for later review. Such reviews were required infrequently, but were found necessary when the two judges identified different reasoning strategies. The most frequent cause for differences in identification of diagnostic reasoning strategy was examinees' use of more than one strategy. For example, the candidate might initiate the diagnostic reasoning process using scheme-inductive inquiry, but resort to deductive reasoning immediately after. Disagreement between the two judges was resolved by discussion until concurrence about the diagnostic reasoning strategy was reached. The final assigned mark reflected the predominant diagnostic reasoning strategy utilized.

A dichotomous score (0 for incorrect answer, 1 for correct answer) was assigned in order to compute the format psychometric properties.

### Data analysis

*Reliability *of the process scores and formats was estimated using Cronbach's alpha coefficient. Item statistics were generated for each item including a discrimination index. Inter-rater reliability of diagnostic reasoning scores was estimated by a Pearson correlation coefficient.

#### Effects of expertise, examination format, and clinical presentations on diagnostic reasoning or 'process score'

A logistic regression analysis was used to determine which of the three independent variables being studied (examination format, expertise, and clinical presentation) had an impact on diagnostic reasoning or 'process score' (the dependent variable). Specifically, the analysis will model the odds of using an 'expert' method of problem-solving, that is scheme-inductive or pattern recognition (in other words, odds of not using hypothetico-deductive reasoning) in relation to the three independent variables of format, expertise and clinical presentation. An expertise effect, which would be expected, will lend evidence of construct validity to the 'process score'. Analysis was carried out using the Stata software system [27].

## Results

### A. Reliability of 'Process Score'

The two judges found it easy to agree on the broad type of strategy used by the subjects (hypothetico-deductive, scheme-directed, and pattern recognition). However, there was less agreement when the same subject used more than one diagnostic strategy. The initial diagnostic reasoning scores resulted in an agreement between the two judges of 0.84.

### B. Reliability and discrimination of examination formats

Both formats demonstrated quite acceptable reliability and discrimination, as per Table 2.

**Table 2:** Cronbach alpha reliabilities and discrimination indices based on question format over all subjects.

Question format	Alpha coefficient	Average disc. index
Multiple-choice	0.76	0.63
Extended-matching	0.66	0.58

### C. Relationship of examination format to cognitive process

The results of the logistic regression analysis are as follows, in Table 3. There was no difference in the odds of using 'expert' methods of problem solving (scheme-inductive or pattern recognition) across the two examination formats (MCQ or EMQ). As would be expected, experts had approximately threefold higher odds of using either of these two problem-solving methods as opposed to novices (p 0.00). There was a negative odd of using scheme-inductive and pattern recognition (-1.55) within the diarrhea and nausea/vomiting clinical presentations (i.e. more likely to use hypothetico-deductive) as opposed to the elevated liver enzymes presentation. Explanation for this lies in the fact that the diarrhea questions were the most complex for both novices and experts (in which case experts and non-experts resorted to hypothetico-deductive reasoning, as has been described in the literature [28]), while the nausea and vomiting questions were complex for the experts especially, given that the experts were gastroenterologists, but the diagnoses for this clinical presentation were 'metabolic' causes of nausea and vomiting.

**Table 3:** Logistic regression of the odds of using an 'expert' process (either pattern recognition or scheme-inductive)

***Independent variable***		***Baseline Level***	***OR (95% CI)***	***p value***
Examination format	Extended-matching	Multiple-choice	-0.59 (-1.73, 0.56)	0.31
Expertise	Expert group	Non-expert group	2.69 (1.64, 3.75)	0.00
Clinical presentation	Nausea and vomiting	Liver enzymes	-1.55 (-2.67, -0.43)	0.01
	Diarrhea	Liver enzymes	-1.55 (-2.67, -0.43)	0.01
	Dysphagia	Liver enzymes	1.11 (-1.21, 1.21)	1.00

### D. Ability of the two formats to evoke higher-order thinking

Table 4 and Table 5 are frequency tables for the Expert and Non-expert Process Scores, across the two examination formats and four Clinical Presentations. They demonstrate that experts utilized either scheme-inductive or pattern recognition more than 90% of the time for both pencil-and-paper examination formats, while non-experts utilized these two reasoning strategies less often than experts, but still greater than 50% of the time for both formats.

**Table 4:** Frequency table for the expert (n = 20) process scores, across two formats and four clinical presentations

Question format	Process score	Liver enzymes	Nausea and vomiting	Diarrhea	Dysphagia	Total
Multiple-choice	1: Hypothetico-deductive	0	3	2	0	5
	2: Scheme	9	4	10	13	36
	3: Pattern recognition	11	13	8	7	39
Total		20	20	20	20	80
Extended-matching	1: Hypothetico-deductive	1	2	1	1	5
	2: Scheme-inductive	8	3	5	14	30
	3: Pattern recognition	11	15	14	5	45
Total		20	20	20	20	80

**Table 5:** Frequency table for the non-expert (n = 20) process scores, across two formats and four clinical presentations

Question Format	Process score	Liver enzymes	Nausea and vomiting	Diarrhea	Dysphagia	Total
Multiple-choice	1: Hypothetico-deductive	6	12	13	6	37
	2: Scheme-inductive	10	2	6	10	28
	3: Pattern recognition	4	6	1	4	15
Total		20	20	20	20	80
Extended-matching	1: Hypothetico-deductive	8	4	14	6	32
	2: Scheme inductive	8	1	4	13	26
	3: Pattern recognition	4	15	2	1	22
Total		20	20	20	20	80

## Discussion

The present study had two major goals. The first was to determine whether the two pencil-and-paper formats studies, the MCQ and EMQ, could in fact assess problem-solving skills. In Table 4 and Table 5, the two pencil-and-paper formats demonstrated high preponderance of scheme-inductive and pattern recognition utilization, in both experts and non-experts, thus suggesting that these question types can potentially elicit higher order clinical reasoning strategies. Another aim was to assess, by using think-aloud protocols, the impact of the alternatives number on psychometric properties and reasoning strategies employed. The logistic regression analysis shown in Table 3 demonstrates that the number of alternatives, in the form of the two examination formats used (MCQ and EMQ), did not exert an independent effect on reasoning strategy utilized. Table 2 demonstrates that both formats had good and comparable psychometric properties.

The first research question of this paper was to investigate whether the examination formats used in this study, the standard five-option Multiple-choice and Extended-matching questions, were capable of testing problem-solving abilities. The observation from the data is that the two formats can potentially evoke more 'expert' methods of diagnostic reasoning processes such as scheme utilization or pattern recognition. Table 4 and Table 5, demonstrate preponderance in both experts (greater than 90%) and non-experts (greater than 50%) of scheme-inductive and pattern recognition utilization in answering the questions. It can be concluded that by evoking these 'expert' methods of clinical reasoning, the two pencil-and-paper formats used in this study have the capability to assess diagnostic higher order thinking, assuming the question stems are constructed with a problem-solving task in mind, as was done in this study.

In regards to the second main research question, the two question formats, with different number of alternatives, did not exert an independent effect on diagnostic reasoning strategy, as shown in the logistic regression analysis (Table 3). Shortening the number of alternatives in the MCQ format to five did not lead to an examinee 'shift' of relying on hypothetico-deductive reasoning. Explanation of this result may be found in the view raised by several authors [6,15] that it is not the examination format, or the number of alternatives, that dictates the cognitive level of the testing, but rather the specific construction of the question stems. We have demonstrated that a well-constructed Multiple-choice question, designed specifically to target problem solving, can achieve the purpose of testing higher order cognitive reasoning. Critics of the Multiple-choice format, who believe that it only tests recall of isolated facts, need to consider altering the construction of the stems rather than the format. That no difference exists between MCQs' and EMQs' relative ability to test for problem-solving may lie in the notion that a person's problem-solving strategy is a trait attribute and not dependent on the item format or number of alternatives. In other words, a given diagnostician will use a given strategy, such as scheme-inductive, on all questions which ask for a problem-solving task (i.e. diagnosis), regardless of the format. A well-created problem-solving question stem will challenge the examinee to use, in many cases, 'expert' (scheme-inductive, pattern recognition) diagnostic reasoning strategies to arrive at an answer, prior to looking at the alternatives. This minimizes the impact of alternatives number on the diagnostic reasoning strategy utilized, and specifically minimizes any shift to hypothetico-deductive reasoning that could have been feared occurring with the smaller number of alternatives presented in the MCQ format. The key is to create the stem with a problem-solving task in mind, and not looking for rote memorization of facts.

While the findings presented do support ongoing use of the MCQ format, there is no denying that the EMQ format has demonstrated superior psychometric properties over the MCQ format in a number of studies mentioned earlier in this paper. Furthermore, in our own study, several non-expert and some expert examinees did comment that Extended-matching questions made it more difficult to go through the list of alternatives prior to answering the question. For examinees relying on hypothetico-deductive reasoning, the Extended-matching format, because of the inherent difficulty of reading through an extended alternatives list, may, at least subjectively, provide a better challenge than the Multiple-choice format.

A significant limitation to the study is the manner in which the cognitive problem-solving process selected by the subjects was ascertained. Thinking aloud was used. After the completion of the examination, subjects were asked to verbally report their thinking method to two judges. The two judges independently noted the cognitive problem solving process the subjects had used in arriving at a diagnosis. Although agreement between the two judges on the process selected was identical in more than 85% of the think-aloud interviews, in the remaining 15% there was disagreement. The cognitive process was then decided by reviewing audiotapes and videotapes, so that 100% agreement could result. In other words, consensus and not initial judgements were used.

## Conclusions

This is the first study that has used this type of think-aloud analysis to directly assess the ability of pencil-and-paper examination formats to test higher order problem solving. The results failed to show a significant difference between the two formats used, but did show that both formats can potentially evoke higher order diagnostic thinking. The results have several potential implications for medical education. Firstly, the results are important to examination construction, by demonstrating direct evidence that problem solving can be tested by pencil-and-paper formats, and specifically change some of the presented misperceptions about the standard five-option MCQ format. Secondly, demonstrating that the two formats can evoke scheme utilization is important. There is evidence [29] that the odds of diagnostic success are greater in examinees using scheme-inductive (and pattern recognition) as opposed to hypothetico-deductive reasoning. However, over and above their potential advantage in problem solving, schemes can be a very powerful tool for knowledge organization in an undergraduate curriculum. In this light, showing that MCQs and EMQs can test for scheme utilization is an important step for medical schools planning to include schemes as a teaching tool in their curricula.

Lastly, this study demonstrates that testing higher order problem solving requires careful attention to question stem rather than question format or number of alternatives. A well-constructed stem will challenge examinees to choose the correct response, potentially using more expert reasoning strategies, prior to examining the alternatives. This has great potential impact on examination writers, who need not feel obliged to provide more than five alternatives, once they have carefully constructed a stem with a problem-solving task in mind.

## Competing interests

The author(s) declare that they have no competing interests.

## Authors' contributions

SC conceived of the study, participated in its design and coordination, and drafted the final manuscript. HM participated in the study conception, design, and revised the initial manuscript draft. PH participated in the study design and performed the statistical analysis. GF participated in the statistical analyses. All authors read and approved the final manuscript.

## Appendix A: The two examination formats

### Format: Multiple-choice question

A 35 year-old woman presents with a one year history of diarrhea. She describes her stools are 10 – 12 profuse, watery, non-bloody bowel movements per day. She is eating well but has lost 7 kg over the last year. She has no abdominal pain. She is unsure if her stools are oily, but they are difficult to flush. She is otherwise perfectly well, with no previous surgeries. She smokes 1/2 pack a day but does not drink alcohol. She has never traveled, camped or drank well water. Her family history reveals an aunt with ulcerative colitis. Examination is unremarkable except for pallor. Stool C & S, O & P and C. difficile are all negative. Laboratory work shows a microcytic anemia (Hb 95, MCV 63), with low ferritin (4), but normal B12 and folate levels.

1) What is the most likely diagnosis for this patient?

A) Celiac disease

B) Crohn's colitis

C) Villous adenoma of rectum

D) Pancreatic insufficiency

E) Bacterial overgrowth ANS:__________

### Format: Extended-matching question

A 33 year-old woman presents with a one year history of diarrhea. She describes her stools as 10 – 12 profuse, watery, non-bloody bowel movements per day. She is eating well, but has lost 9 kg over the last year. She has no abdominal pain. She sometimes sees oil droplets in her stool, and they are very difficult to flush. She had surgery for stomach ulcers at age 20, and had repeat surgery five years later for "bile gastritis". She is otherwise healthy. She smokes 1/2 pack per day but does not drink alcohol. She has not drank well water, and has not traveled or gone camping recently. Her family history is significant for two cousins with Crohn's disease. Examination is unremarkable. Stool C & S, O & P and C. difficile are all negative. Her CBC shows a macrocytic anemia (Hb 108, MCV 110) with a normal ferritin, but low B12 and elevated folate levels.

Select the most likely diagnosis from the list below: __________________

A) Bacterial overgrowth

B) Celiac disease

C) Collagenous colitis

D) Crohn's colitis

E) Crohn's ileitis

F) Colonic carcinoma

G) Factitious diarrhea

H) Giardiasis

I) Ischemic colitis

J) Irritable bowel syndrome

K) Lactose intolerance

L) Pancreatic insufficiency

M) Shigella dysentery

N) Villous adenoma of rectum

O) Viral gastroenteritis

## Pre-publication history

The pre-publication history for this paper can be accessed here:


